# Successful Crossover Bypass Using a Lateral Femoral Circumflex Artery as an Outflow Vessel for Indirect Revascularization in Critical Limb Ischemia: A Case Report

**DOI:** 10.3400/avd.cr.22-00059

**Published:** 2022-12-25

**Authors:** Kei Akiyoshi, Mamoru Arakawa, Harunobu Matsumoto, Koichi Adachi, Hiroko Nakata

**Affiliations:** 1 Department of Cardiovascular Surgery, Yokosuka Uwamachi Hospital, Yokosuka, Kanagawa, Japan; 2 Department of Vascular Surgery, Saitama Medical University Hospital, Saitama, Saitama, Japan

**Keywords:** critical limb ischemia, lateral femoral circumflex artery, sequential bypass

## Abstract

A 78-year-old man presented with severe stage 3 (Fontaine IV, Rutherford 5, W1 I3 FI0) right limb ischemia. Although his artery was completely occluded from below the right external iliac to the popliteal artery, collateral circulation from the right lateral femoral circumflex artery was well developed and supplied the lower extremity arteries. We selected an uncommon crossover bypass strategy with the left common femoral artery to the right lateral femoral circumflex artery to improve lower extremity perfusion via indirect revascularization. Bypass using the lateral femoral circumflex artery as an outflow is an option for patients with major lower extremity artery occlusions.

## Introduction

Lower extremity bypass is recommended for long occlusive arterial lesions, such as those characterized as Inter-Society Consensus for the Management of Peripheral Arterial Disease (TASC) D.^1)^ Nevertheless, the selection of inflow and outflow vessels during bypass planning is often challenging in patients with critical limb ischemia (CLI) considering that many have occlusion of the major target vessels of the lower extremities. In this study, we report the successful treatment of CLI with a right toe refractory wound using an uncommon strategy of crossover bypass from the left common to the right lateral femoral circumflex artery (LFCA) and LFCA’s branch.

## Case Report

**Figure 1:**
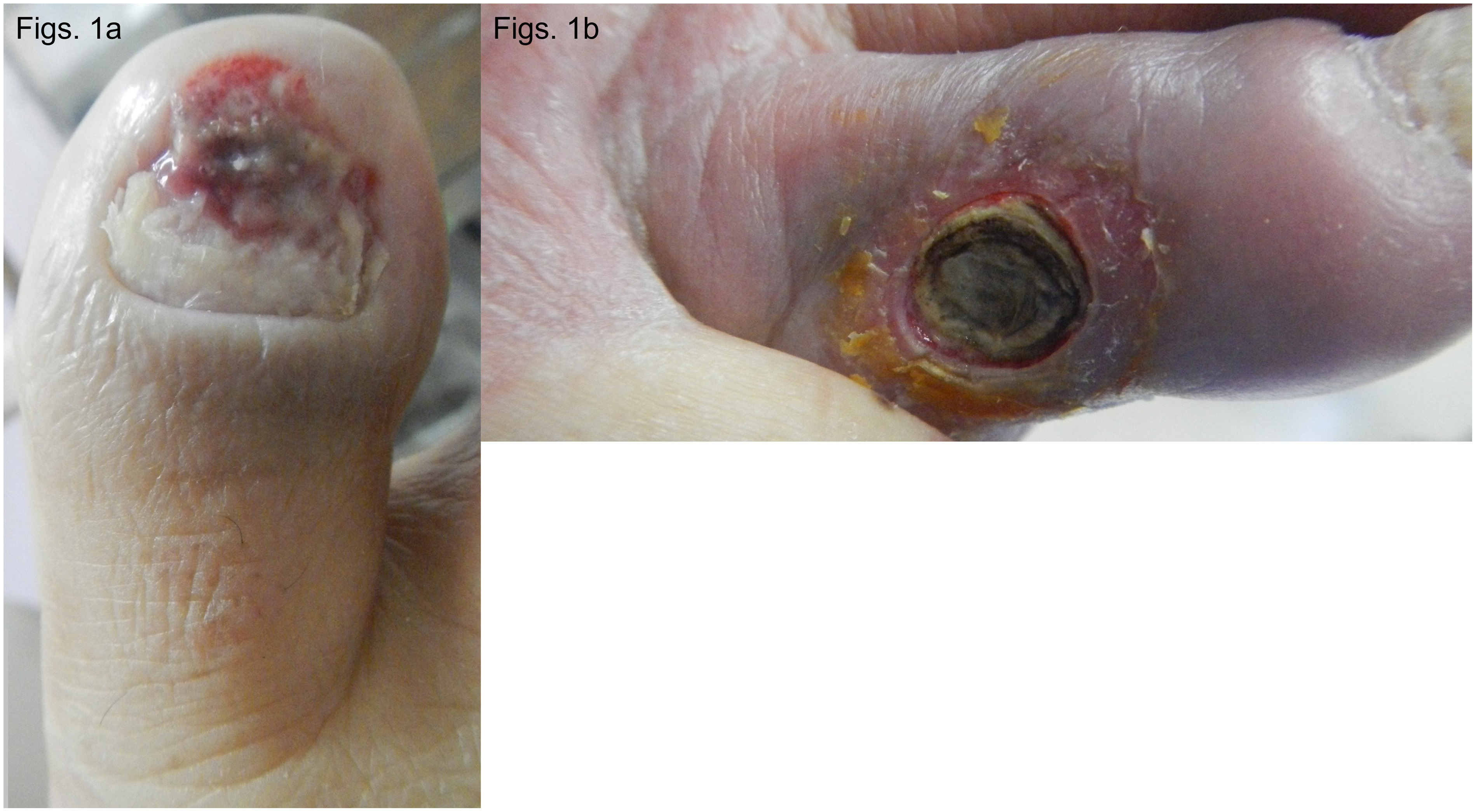
Fig. 1 Preoperative findings of refractory ulcers of the first and fourth right toes of the patient. (**a**) Right first toe. (**b**) Right fourth toe.

A 78-year-old man with a past medical history of ischemic heart disease, hypertension, atrial fibrillation, hyperlipidemia, and postabdominal aortic aneurysm repair was referred to our hospital for the treatment of refractory ulcers of his right first and fourth toes. The patient had a history of refractory ulcers for more than 1 year (**Figs. 1a** and **1b**). His current medications included clopidogrel sulfate 75 mg, rivaroxaban 15 mg, rosuvastatin calcium 5 mg, azilsartan 40 mg, and bisoprolol fumarate 1.25 mg. Lower extremity arteries were not palpable below his femoral arteries, excluding the left femoral artery. His ankle–brachial pressure index was unmeasurable on the right side and 0.53 on the left side. Dorsal, medial plantar, and lateral plantar skin perfusion pressure (SPP) values were 7, 3, and 9 mmHg, respectively. The patient was clinically diagnosed with severe right limb ischemia stage 3 (Fontaine IV, Rutherford 5, W1 I3 FI0).

**Figure 2:**
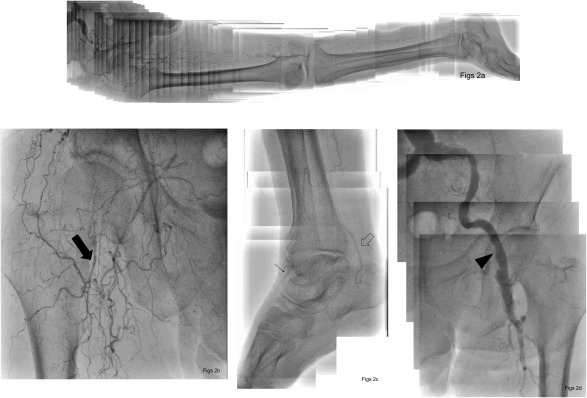
Fig. 2 Preoperative angiography. (**a**–**c**) The lower right limb had a very long occlusive lesion below the external iliac artery. However, collateral circulation from the LFCA (➡) was well developed, and the distal PTA (⇨) and dorsal artery (→) were enhanced via collateral circulation. (**d**) The left femoral artery (▶) was patent.

LFCA: lateral femoral circumflex artery; PTA: posterior tibial artery

Subsequent angiography of the lower right limb revealed a very long occlusive lesion below the external iliac artery, which involved the profunda femoris artery and superficial femoral artery (SFA). Nevertheless, collateral circulation from the LFCA was well developed, and the distal posterior tibial artery (PTA) and dorsal artery were enhanced via collateral circulation, with some collateral vessels from the LFCA connecting the PTA and dorsal artery directly (**Figs. 2a**–**2c**). Unlike the lower right limb, the lower left limb had patent external iliac and common femoral arteries. However, a long occlusive lesion below the proximal SFA and distal PTA was enhanced via collateral circulation (**Fig. 2d**).

To improve lower limb blood flow and allow for additional bypass using this crossover bypass as an inflow vessel in the future if this procedure proves insufficient for healing the right foot, we selected an uncommon bypass strategy in which the left common femoral artery was used to enhance blood flow to the right foot via rich collateral circulation through the LFCA considering the patient’s general status and grafts availability. The entire procedure was performed under general anesthesia. The great saphenous vein (GSV) was harvested from the right femur using a standard bridging technique. The left and right femoral arteries, right profunda femoris artery, and right LFCA were exposed using longitudinal groin incisions of approximately 6 cm on the left and right sides. The right LFCA originated directly from the common femoral artery. Next, it was noted that the common femoral artery bifurcated into the SFA and deep femoral artery (DFA) in the patient. A GSV was tunneled through the suprapubic subcutaneous layer from one groin incision to the other and used as a reversed GSV. End-to-side proximal anastomosis of the left common femoral artery was performed using a parachute technique and 6-0 polypropylene (Matsuda, Tokyo, Japan) sutures. Distal anastomosis to the LFCA and its branch was performed using the parachute technique and 7-0 polypropylene (Matsuda, Tokyo, Japan) sutures. Intraoperative graft flow measured using a VeriQ system (MediStim, Oslo, Norway) was determined to be sufficient for completing the operation because the measured value was 140 mL/min, and the typically used cutoff value for graft flow is 20 mL/min.^2)^

**Figure 3:**
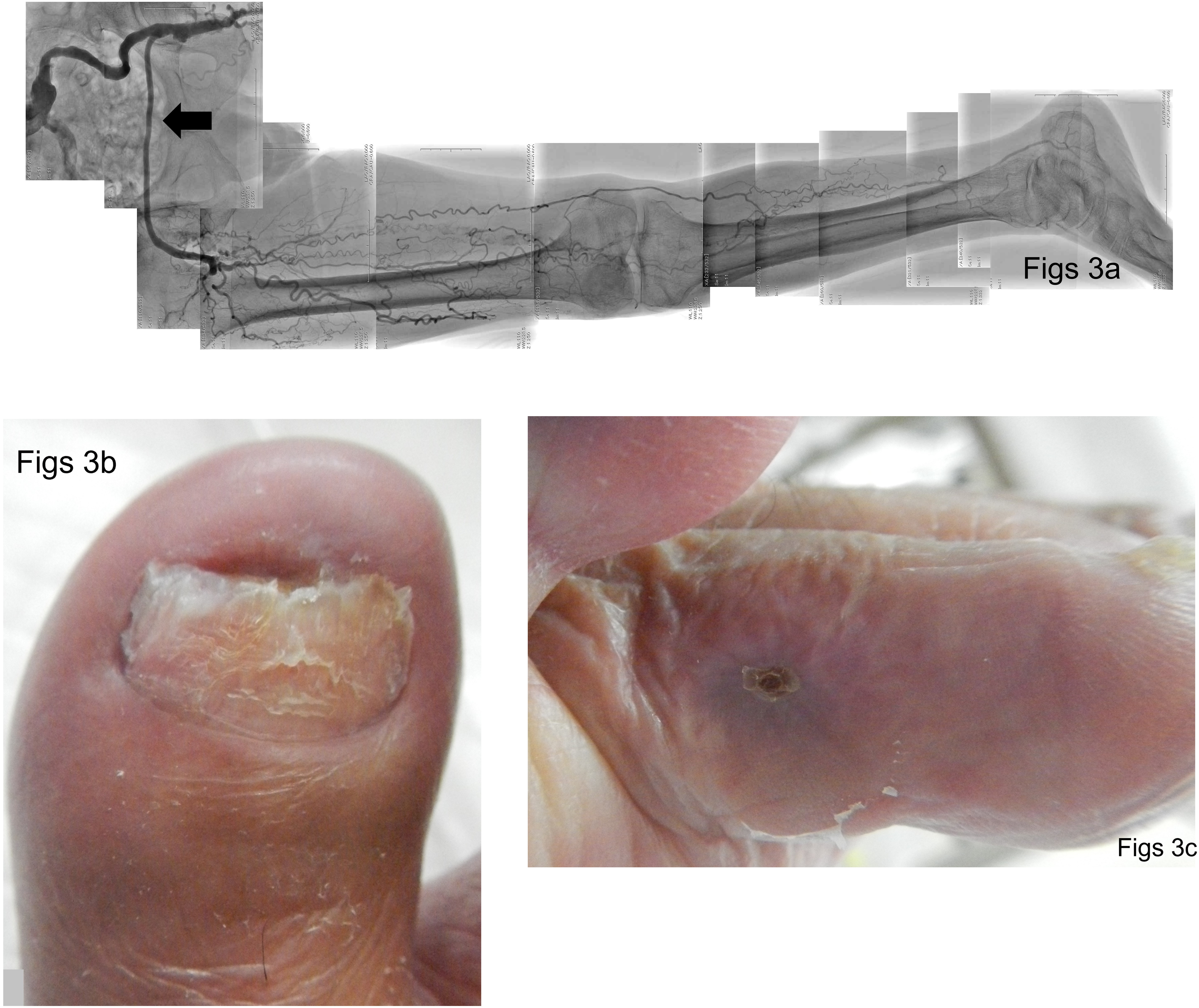
Fig. 3 Postoperative enhanced computed tomography and toes in 6 months. (**a**) The graft (➡) from the left common femoral artery to the right LFCA was patent. (**b**, **c**) The ulcers visually disappeared 6 months postsurgery.

The postoperative course of the patient was uneventful. Postoperative SPP improved (dorsal: 37 mmHg, medial plantar: 22 mmHg, lateral plantar: 26 mmHg), and his right foot temperature normalized. Postoperative angiography showed a patent bypass graft and good blood flow to the foot via collateral circulation (**Fig. 3a**). The patient was discharged on postoperative day 11, taking all preoperative drugs. His ulcers shrunk gradually and visually disappeared at 6 months postsurgery (**Figs. 3b** and **3c**). Presently, the bypass graft is patent at 5 years postsurgery.

LFCA: lateral femoral circumflex artery

## Discussion

Here, we report the successful use of an uncommon left femoral artery to the right LFCA crossover bypass procedure. Our patient had a long occlusion below the right external iliac artery. Furthermore, percutaneous transluminal angioplasty was not suitable, and lower extremity bypass was challenging due to the difficulty of inflow vessel selection. Although profundaplasty was considered another treatment option, the presence of a very long occlusive lesion in the area of runoff raised concerns about limited graft patency.^3)^ Initially, lower extremity perfusion was indirectly improved via crossover bypass. This strategy was undertaken to allow a bypass procedure with the implanted graft on the right side as an inflow if ulcers failed to heal postsurgery. Although the bypass flow was indirect, it improved lower extremity perfusion, resulting in the patient’s CLI-induced toe ulcer disappearance. To our knowledge, there are few reports of indirect revascularization through the LFCA that contributed to ulcer healing in a CLI patient.

In patients with long occlusive arteries that include a major bypass target, selecting bypass inflow and outflow targets is challenging. The current literature suggests that obtaining direct pulsatile flow by direct revascularization to the foot is the main objective of CLI revascularization. However, as observed in this case, direct revascularization is sometimes difficult.^4,5)^ By contrast, some studies indicated that the presence of collateral vessels, patent pedal arch, or peroneal distal branches may facilitate limb salvage, despite the absence of revascularization.^6)^ In our case, collateral arteries were well developed from the LFCA to the distal PTA and dorsal artery via peroneal distal branches. Although surgeons may be hesitant to select a branch as an outflow target, we used the left femoral artery and LFCA as inflow and outflow targets, respectively, which functioned as a well-developed collateral source in the patient. Fortunately, the patient’s right toe ulcers shrunk and visually disappeared after the bypass. This case indicates that indirect revascularization is a treatment option in patients with well-developed collateral circulation when direct revascularization is difficult.

Two previously reported cases described using the LFCA as an outflow target in patients with total occlusion of all major femoral arteries.^7,8)^ In one report, the LFCA was used for the end outflow. In another, the LFCA was used as a relay point for sequential bypass. The authors indicated that the LFCA could serve as a suitable outflow route. However, care should be taken to avoid complications due to LFCA fragility and the propensity for spasms.^7,8)^ While 5 years have passed after the surgery, the graft remains patent and supplies sufficient blood flow to the foot via collateral circulation from LFCA. The LFCA is a possible outflow target, and the crossover bypass graft itself is a potential inflow target if future lower extremity bypass to posttibial or dorsal arteries is required.

When using the LFCA as an outflow target, the LFCA variance should be considered. The anatomical variance of the LFCA has been reported.^9,10)^ The LFCA originates from the DFA (most frequently, in 70%–80% cases), followed by the common femoral artery (10%–20% cases), and proximal to the external iliac artery (6% cases). In our patient, the LFCA originated directly from the femoral artery and was well developed, providing collateral circulation.^9,10)^ Variance contributes to the development of collateral circulation. Before using the LFCA as an outflow target, confirmation of variance via computed tomography or angiography is necessary.

## Conclusion

To salvage a lower limb in a patient with long occlusive major arteries in a lower extremity, indirect revascularization is a treatment option. Additionally, the LFCA is a possible outflow target vessel in crossover bypass if it is a sufficient collateral source.
